# Quantitative 3D OPT and LSFM datasets of pancreata from mice with streptozotocin-induced diabetes

**DOI:** 10.1038/s41597-022-01546-5

**Published:** 2022-09-10

**Authors:** Max Hahn, Christoffer Nord, Pim P. van Krieken, Per-Olof Berggren, Erwin Ilegems, Abbas Cheddad, Ulf Ahlgren

**Affiliations:** 1 Umeå Centre for Molecular Medicine, Umeå University, Umeå, Sweden; 2 The Rolf Luft Research Center for Diabetes and Endocrinology, Karolinska Institutet, Stockholm, Sweden; 3 Dept. of Computer Science, Blekinge inst. of technology, Karlskrona, Sweden

**Keywords:** Optical imaging, Biologics, Diabetes, Islets of Langerhans, Preclinical research

## Abstract

Mouse models for streptozotocin (STZ) induced diabetes probably represent the most widely used systems for preclinical diabetes research, owing to the compound’s toxic effect on pancreatic β-cells. However, a comprehensive view of pancreatic β-cell mass distribution subject to STZ administration is lacking. Previous assessments have largely relied on the extrapolation of stereological sections, which provide limited 3D-spatial and quantitative information. This data descriptor presents multiple *ex vivo* tomographic optical image datasets of the full β-cell mass distribution in mice subject to single high and multiple low doses of STZ administration, and in glycaemia recovered mice. The data further include information about structural features, such as individual islet β-cell volumes, spatial coordinates, and shape as well as signal intensities for both insulin and GLUT2. Together, they provide the most comprehensive anatomical record of the effects of STZ administration on the islet of Langerhans in mice. As such, this data descriptor may serve as reference material to facilitate the planning, use and (re)interpretation of this widely used disease model.

## Abstract (summary)

**Table:** 

Measurement(s)	Fluorescent antibody staining of Insulin and GLUT2 in whole mouse pancreata
Technology Type(s)	Optical Projection Tomography • Light Sheet Fluorescence Microscopy (Ultramicroscope)
Factor Type(s)	mouse genotype • Streptozotocin dosage
Sample Characteristic - Organism	Mus musculus

## Background & Summary

**Fig. 1:**
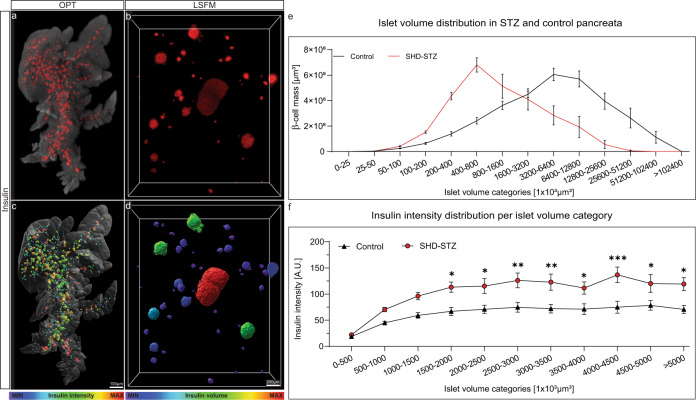
Examples of OPT and LSFM based data visualization and quantification. The datasets included in this data descriptor can be used for a multitude of assessments pertinent to STZ-induced diabetes in mice. This includes a range of quantitative and 3D-spatial analyses (volumes, signal intensities, numbers, shapes, distribution patterns, etc.) of insulin and GLUT2 stained islets of Langerhans subject to different modes of STZ administration and at different time points. (**a**,**b**) Images of maximum intensity projections based on pancreatic volumes from control C57BL/6 mice showing the distribution of insulin labelled islets of Langerhans (red) in the pancreatic splenic lobe imaged by OPT (**a**) and high-resolution LSFM image (**b**). (**c,d)** The same specimen as in (**a,b**) in which the intensity of the signal intensity has been colour coded. Blue colour corresponds to low and red to high signal intensity, respectively. (**e**) Graph illustrating the possibility to study statistically assess spatial distribution patterns subject to STZ treatment. (**f**) Graph illustrating the possibility to statistically assess staining intensities of islets of different size categories subject to STZ administration. Data in (**e,f**) is based on pancreata 2 weeks post SHD administration). Scale bar in (**c**,**d**) corresponds to 1 mm and 200 µm in (**a,b**) respectively. Data is presented as averages and error bars represent SEM. Significance in graph (**f**) was tested using a One-way ANOVA. * represents *P* ≤ 0.05, ** represents *P* ≤ 0.01 and *** represents *P* ≤ 0.001.

The pancreas is a key organ in the development of diabetes mellitus. The gland has two primary functions in that it produces digestive enzymes for breakdown of food and endocrine hormones for maintaining blood glucose homeostasis. The endocrine component, organised into the islets of Langerhans, consist of five hormone-producing cell types, of which the insulin producing β-cells is the most abundant (around 80% in mice). A major challenge for assessments of changes in the mass and phenotype of the pancreatic endocrine cells is the scattered distribution of the islets in the pancreatic parenchyma. While the islets normally are present by the thousands in the murine pancreas^1,2^, they constitute only a fraction (around 1–2%) of the total pancreatic volume. Further, they display significant heterogeneities in size both within and between the primary lobes of the pancreas^3,4^. Subsequently, the possibility to obtain a comprehensive view of changes in β-cell mass and function that reflects the condition of the entire organ are exceedingly difficult to perform by extrapolation of stereological sections. Mesoscopic optical imaging techniques such as Optical Projection Tomography (OPT)^2,5,6^ have made it possible to characterise the β-cell mass (BCM) in 3D throughout the entire volume of the pancreas at islet level resolution in large cohorts of animals (see Fig. 1, dataset 1 and 2^7,8^). Moreover, recent advancements in light sheet fluorescent microscopy (LSFM) allows for subsequent assessments of the same samples without additional processing, yielding results down to single cell resolution (dataset 3^8^).

**Fig. 2:**
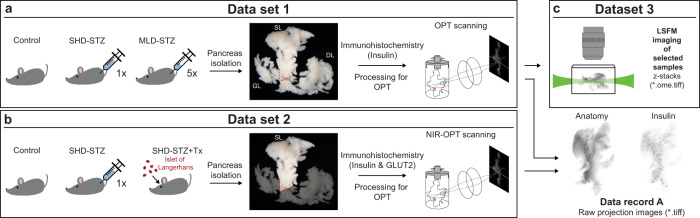
Schematic illustration of experimental setup and data generation. (**a**) Animals were subjected to different STZ treatment regimens and the pancreata were collected over 3 weeks. The pancreata were subsequently divided into the three primary lobular compartments (splenic lobe (SL), duodenal lobe (DL) and gastric lobe (GL)) prior to whole mount immunohistochemistry (insulin-594 and GLUT2-680), agarose embedding and tissue clearing for OPT-scanning. dataset 1 consist of control, single high dose (SHD) and multiple low doses (MLD) at 1-, 2- and 3-weeks post STZ administration (n = 5, n = 3–5 and n = 3–5, respectively per time point). (**b**) Animals were subjected to a single high dose STZ treatment and recovered from hyperglycemia by transplanting islet of Langerhans to the anterior chamber of the eye (SHD + Tx). dataset 2 consists of data from pancreatic splenic lobe (SL) only from control samples (n = 7), SHD (n = 8) and SHD + Tx (n = 4) 4 weeks post STZ administration. (**c**) dataset 3 consists of LSFM image data and is generated based on a selection of representative samples from dataset 1.

Streptozotocin (STZ) administration is frequently applied within academia and pharmaceutical industry to generate models of hyperglycemia and diabetes (≈68,000 articles in relation to “Streptozotocin diabetes” listed in PubMed as of June 2022). The models are simple to produce, have a predictable time course of disease progression and can be modulated to mimic primary features of human diabetes. STZ is a glucose analogue particularly toxic to the pancreatic β-cells^9,10^. It enters the β-cell via the low affinity glucose transport type 2 (GLUT2), which has a vital role in glucose sensing, and thus in the insulin secretion process^11^. When administered in a single high dose (SHD), it results in hyperglycaemia and β-cell ablation, whereas administrated in multiple low doses (MLD), STZ induces slowly increasing hyperglycaemia and often induces insulitis^9,12,13^. Pancreata from STZ-treated mice were compared to pancrerata vehicle control animals (SHDvCtrl and MLDvCtrl), as well as non-treated control (Ctrl). Recently, we investigated how different modes of STZ administration affect BCM and β-cell functionality in the entire mouse pancreas^14^ (for experimental design, see Fig. 2).

## Methods

### Animals, STZ administration and organ isolation

The data presented in this data descriptor was acquired for Hahn *et al*.^14^ and all experiments were performed following the European Union guidelines for care and use of animals in research. All procedures were approved by the Animal Review Board at the Court of Appeal of Northern Norrland and of Northern Stockholm. Streptozotocin (STZ, Sigma-Aldrich) dissolved freshly in 0.1 M sodium citrate buffer (pH 4.5) was administered to 8-week-old male C57BL/6 J mice by intraperitoneal (i.p.) injection, either as a single high dose (SHD, 150 mg/kg) or as multiple low doses (MLD, 50 mg/kg over 5 consecutive days). For data reliability, untreated control mice were compared with mice receiving an i.p. injection of the vehicle solution only (0.1 M sodium citrate, pH 4.5). No difference in BCM, islet number or blood glucose levels could be detected (see Hahn *et al*.^14^, suppl. Figure 2). Glucose measurements were regularly performed from tail vein blood with OneTouch (LifeScan, USA) or Accu-Chek (Roche, Switzerland) glucometers until death (see Tables S1 and S2). Animals were killed by cervical dislocation and pancreata from diabetic groups of SHD or MLD and healthy control groups were isolated at 1-, 2- and 3-weeks post administration of STZ (n = 3–5, n = 3–5 and n = 5 respectively). Harvested pancreata were fixed in 4% paraformaldehyde (PFA, Sigma Aldrich) for 2 h, washed in 1x PBS and divided into the splenic, gastric, and duodenal lobular compartments^3,4^ (see also Fig. 2) before processing for whole mount immunohistochemistry and 3D imaging.

To delineate the long-term effects of hyperglycemia on GLUT2 expression, on β-cell function and islet size distribution generated data from islet transplantation experiments (see Fig. 2, dataset 2^8^) was performed as previously described^15^. In short, pancreatic islets of Langerhans were obtained from healthy (normoglycemic) mice with the same genetic background via collagenase treatment. SHD treated animals with the highest blood glucose levels 4 days post-STZ administration were then transplanted with 100–150 islets per animal into the anterior chamber of the eye under isoflurane anaesthesia to revert hyperglycemia. Once the SHD treated (n = 8) and islet transplanted cohort (SHD + Tx, n = 4) reached normoglycemic levels to the control (n = 7), organs of all animal cohorts were harvested (28 days post STZ administration, see above).

### Pancreas processing, whole mount immunohistochemistry and tissue clearing

Tissue processing, staining procedure, and preparation for OPT/LSFM imaging was performed as described^14^. In brief, isolated and fixed pancreata were separated into the main lobes (SL, GL and DL respectively, see Fig. 2) permeabilized by freeze/thawing cycles, bleached to reduce autofluorescence, stained with primary and secondary antibodies, mounted in a cylinder of low melting point agarose, dehydrated with methanol and made transparent by matching the refractive index of proteins, lipids, and other cellular components with a 1:2 mixture of benzyl alcohol and benzyl benzoate (BABB), respectively. All specimens were blinded and randomized after organ harvest for all downstream processes. Primary antibody used was guinea pig anti-insulin (DAKO A0594, dilution 1:500) and secondary was goat Alexa 594 anti-guinea pig (Molecular Probes, A11076, dilution 1:500). For co-expression assessments of insulin and GLUT2 (see Figs. 1 and 2, dataset 2^8^), pancreata were in addition to insulin labelled with primary rabbit anti-GLUT2 (Millipore, 07-1402-l, dilution 1:500) and secondary IRDye 680RD goat anti-rabbit (Licor, 926–68071, dilution 1:500).

### 3D imaging: Optical projection tomography (OPT) and Light sheet fluorescent microscopy (LSFM)

OPT scanning of pancreatic specimen (dataset 1)^7^ was performed as described^14^ using a Bioptonics 3001 OPT scanner (SkyScan, Belgium) with varying exposure times (see folder “Metadata for all groups” for exposure times) of Insulin staining (filter set “insulin”: Ex:560/20 nm, Em.:610 nm LP) and autofluorescence (filter set “anatomy”: Ex: 425/20 nm, Em.:475 nm LP). The image data was generated using SkyScanner 3001 (v1.3.13, SkyScan). Samples from co-expression experiments (dataset 2)^8^ were scanned in our custom build Near Infrared-OPT setup^16^ using LabVIEW (v20.0f1) to retrieve image data. For comparison of intensities in 3D,all images in dataset 2 were generated using equal exposure times of Insulin staining (filter set: Ex: HQ 565/30 nm, Em: HQ 620/60 nm, exp. t = 4000 ms), GLUT2 staining (filter set: Ex: HQ 665/45 nm, Em: HQ 725/50 nm, exp. t = 8000 ms) and endogenous fluorescent anatomy (filter set: Ex: 425/60 nm Em: LP 480 nm, exp. t = 500 ms).

Additional high-resolution scans (dataset 3)^8^ of volumes of interest from representative pancreata that were OPT scanned (see above) were reimaged in a LaVision biotech 2^nd^ generation UltraMicroscope (LaVision BioTec BmbH, Germany) with a 1x Olympus objective (Olympuse PLAPO 2XC) coupled to an Olympus MVX10 zoom body, providing between 0.36x and 6.3x magnification with a lens corrected dipping cap MVPLAPO 2x DC DBE objective. Samples mounted in low melting point SeaPlaque Agarose (39346-81-1, Lonza) were trimmed in BABB to fit the LSFM sample holder. Scans were acquired using 6.3 x magnification, which rendered a pixel size of 0.48 µm in x and y dimensions. Depending on the scan locations, the exposure time was 120–300 ms, light sheet-width was between 10–20% with 3.78 µm thickness (NA of 0.14) with a z-step size of 5 µm. Image data acquisition was performed using ImSpectorPro (version 5.0.164, LaVision BioTec GmbH, Germany). Representative islets of Langerhans with different sizes and locations in the gland were chosen based on 3D rendered OPT datasets.

### Image processing, reconstruction, and 3D volume rendering

Insulin based projection views retrieved from the Bioptonics 3001 scanner (dataset 1)^7^ and volumetric assessments on β-cell volumes retrieved from the custom build NIR-OPT scanner (dataset 2)^8^ were first processed with a contrast limited adaptive histogram equalization (CLAHE) algorithm^2^, with a tile size of 64 × 64 to increase the signal-to-noise ratio for downstream islet segmentation. Secondly, a discrete Fourier transform alignment (DFTA)^17^ was performed to align opposing projection images to the same axis of rotation of a sample. However, for combined assessments of insulin and GLUT2 expression (dataset 2)^8^ and analysis of the effect of STZ on GLUT2 staining intensity in β-cells, the CLAHE normalisation routine was not implemented. Reconstruction of OPT projection views to tomographic sections was performed using a filtered back projection algorithm in the NRecon software (V1.6.9.18, Bruker microCT, Belgium) with ring artefact correction set to 4. Resulting tomographic sections (*.bmp and *.tif, datasets 1 & 2, Record B1 and B2) and raw z-sectional images (*.ome.tif, dataset 3, Record B) generated with the Ultramicroscope II (LSFM) of each channel were converted with Imaris converter (Bitplane, UK) and the subsequential *.ims files of each channel for each sample were incorporated into one imaris file. Individual insulin positive islet volumes and lobular anatomies were quantified using an automated surfacing algorithm within the Imaris software (version 9.3.1, Bitplane, UK). Surface segmentation was performed using the ‘background subtraction’ function in Imaris with varying thresholding between samples. Individual threshold values for islet volume segmentation are displayed in Supplementary Tables 3 and 4 for dataset 1 and 2 respectively. Threshold values for islet volume segmentation between the administration groups were plotted and compared to each other (Supplementary Fig. 1). Surfaced islet volumes were arbitrarily categorized into small (<1 × 10^6^ µm^3^), medium (1–5 × 10^6^ µm^3^) and large (>5 × 10^6^ µm^3^) islets of Langerhans as previously described^18^. Volumes of 10 voxels or less were filtered out from quantification datasets (dataset 1 & 2) to avoid inclusions of artefacts in the data analysis.

## Data Records

In this data descriptor, we present the datasets underlying this study, which are stored on the DRYAD data repository, dataset 1–3^7,8^, and examples from each dataset to facilitate initial viewing and downloading, Sample dataset^19^.

**Dataset 1**: Volumetric and 3D-spatial OPT assessments of BCM distribution from pancreatic compartments (splenic lobe (SL), duodenal lobe (DL), gastric lobe (GL)) in a SHD and MLD diabetic mice, 1-, 2- and 3-weeks post injection in comparison to vehicle controls (SHDvCtrl, MLDvCtrl) and untreated controls (Ctrl) with corresponding blood glucose levels and body weights (Table S1)^7^.

**Dataset 2**: OPT analysis of BCM and GLUT2 3D expression intensities in pancreatic splenic lobes from SHD induced hyperglycaemia mice, in which glycemia was restored by islet transplantation (SHD + Tx). Pancreata were collected 28 days post administration of STZ and compared to vehicle control and SHD positive control with corresponding blood glucose levels and body weights (Table S2)^8^.

**Dataset 3**: High-resolution assessments of islet morphology using LSFM from representative samples from dataset 1^8^.

**Fig. 3:**
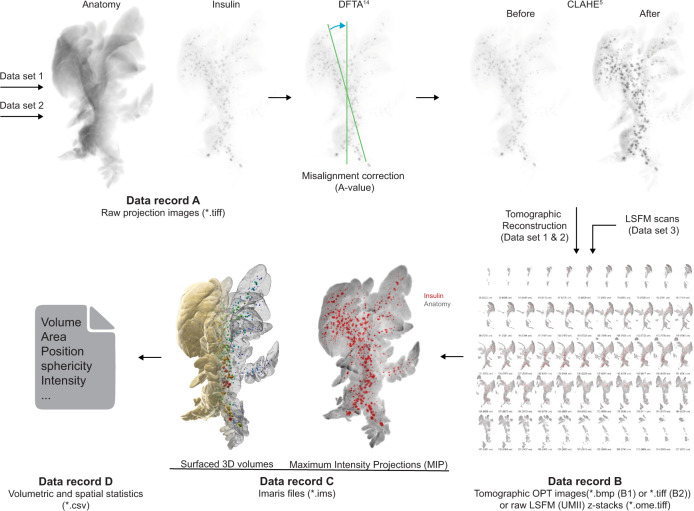
Data processing pipeline. Data record A from dataset 1 (Data record A, Data citation 1) and 2 (Data record A, Data citation 5) were processed using a set of in-house developed post-scanning computational scripts, DFTA (uniform alignment values) and CLAHE (equalizing the contrast of the insulin labelled islets) prior to reconstruction into tomographic images (image processing package “DSPOPT”, including DFTA (“A-value” tuning) and CLAHE can be found: https://github.com/ARDISDataset/DSPOPT). Data record B also include LSFM z-stacks from dataset 3 (Data citation 2, 6 and 9). The tomographic images converted to Imaris native.ims files were analysed (Data record C, 3, 7 and 10). Volumetric and spatial statistics was extracted in Imaris (Data record D, Data citation 4 and 8).

Each dataset is subdivided into data records, based on the image processing pipeline (see Fig. 3). The provided raw projection views (data record A, datasets 1 & 2, data citation 1 & 5) were generated by an in house build near infrared -OPT scanner^16^ as *.tiff files. For data record B, tomographic 2D image datasets were processed and reconstructed into tomographic sections (datasets 1 & 2, data citation 2 and 6). Data record B further includes unprocessed LSFM generated sections (Dataset 3, data citation 9). For data record C, Z-sections from OPT and LSFM imaging were transformed into Imaris (*.ims) files for assessments of spatial and quantitative features of BCM distribution (dataset 1,2 & 3, data citation 3, 7 & 10). The resulting quantitative data were extracted from Imaris as Excel sheets (data record D, data citation 4 & 8) comprising numerical data on islets volumes, staining intensities, and islet sphericity, together with data on the pancreatic lobular anatomy. Jointly, the presented datasets may facilitate the planning, execution, and evaluation of a range of research undertakings pertaining to STZ-induced diabetes in rodents.

**Fig. 4:**
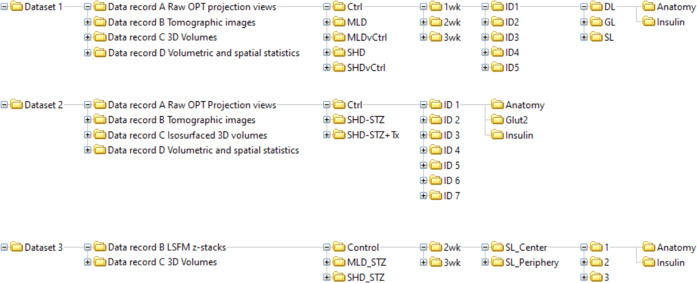
Schematic illustration of data folder tree. Schematic illustration depicting the sub-organization of the datasets incorporated in the data descriptor including data records A-D, treatment groups, time points post STZ administration, sample IDs, imaging channels and scan location (LSFM).

A schematic image of the organised data tree is displayed in Fig. 4. The data records describe the raw, processed (tomographic reconstructions), end point image datasets and quantitative/spatial data of the full BCM distribution in STZ-induced diabetic mice (SHD, MLD, SHD + Tx) and their healthy (C57BL/6) controls at 1-, 2- and 3-weeks post-administration, as well as vehicle controls (SHDvCtrl and MLDvCtrl). Note, the MLD group at the 1-week time point did not have any diabetic animals but were still included in the dataset.

### Data record A

Raw projection datasets generated by OPT can be found in ‘Data record A Raw OPT projection views’ (Data record A Raw OPT projection views.zip, Data citation 1 and 5). Each individual scan is supported by a log file in *.txt format including scanning parameters such as exposure times or rotation steps. The individual image files are titled to indicate experimental group, age post-administration, animal ID, pancreatic lobe (splenic (SL), duodenal (DL) or gastric (GL)), channel (Insulin or Anatomy or for dataset 2 GLUT2) and step rotation number (1 step = 0.9 degrees of rotation) of projection image, e.g., ‘SHD_2wk_ID3_SL_Insulin_0398.tif‘.

### Data record B

Data generated by tomographic reconstruction of OPT processed data (Axis of rotation, DFTA and CLAHE) can be found in ‘Data record B Tomographic images’ (Data record B Tomographic images.zip, Data citation 2 and 6), as well as raw generated LSFM z-sections as ‘Data record B LSFM z-stacks’ (Data record B LSFM z-stacks.zip, Data citation 9). The individual image sections of dataset 1 and 2 are annotated to indicate experimental group, age post-administration, animal ID, pancreatic lobe (SL, DL, GL respectively), channel (Insulin or Anatomy, or GLUT2 for dataset 2) and sequential z-stack number, e.g., ‘Ctrl_1wk_ID4_DL_Anatomy_0554.bmp’. Each LSFM section in record B in dataset 3 indicates experimental group, age post-administration, location of scanned volume (periphery or centre of the pancreatic splenic lobe), scan ID, channel (Insulin or Anatomy) and z-stack number, e.g., ‘MLD_STZ_2wk_SL_Center_2_Insulin_Z0003.ome.tif.

### Data record C

Reconstructed data converted to *.ims format with 3D iso-surfaced volumes can be found in ‘Data record_C_Isosurfaced volume files’ (Data record_C_ Isosurfaced volume files.zip, Data citation 3 and 7) and for Dataset 3 Volume files (Data citation 10). The files contain (for datasets 1 and 2) iso-surfaces of islets of Langerhans based on the Insulin channel and of the lobular anatomy based on the anatomy channel, and 3D-volumes of the above-mentioned structures (dataset 3). They are annotated to indicate experimental group, age post-administration, animal ID and pancreatic lobe (splenic (SL), duodenal (DL) or gastric (GL)), e.g., ‘Ctrl_3w_ID4_SL.ims.

### Data record D

Resulting quantitative data of processed 3D OPT volumes were retrieved from Imaris and can be found in ‘Data record D volumetric and spatial statistics’ (Data record D volumetric and spatial statistics.zip, Data citation 4 and 8). Reconstructed OPT scans produce isotropic voxels, which allows for reliable quantification, whereas LSFM scans in general have a distortion in the z-axis, as do the Ultramicroscope II used in this study due to the generation of the light sheet being two cones overlapping into each other rather than two parallel lines. Therefore, quantitative data is given for OPT data only. The OPT based Excel sheets are raw extracted numerical information. File titles indicate experimental group, age post-administration, animal ID, pancreatic lobe (splenic (SL), duodenal (DL) or gastric (GL)) and channel information, e.g., ‘Ctrl_3w_ID4_DL_anatomy.csv’. The csv files are subdivided into multiples files, each displaying a different parameter for the individual islets:

- Volume
- Area
- Position
- Sphericity
- Intensity (centre, min, max, mean, median, sum)
- Dimensional (x,y,z) diameter
- Centre of homogenous mass
- Distance to image border
- Ellipsoid axis
- Ellipticity (oblate/prolate)
- Number of triangles
- Number of vertices
- Number of voxels

Supplementary Tables 3 and 4 display meta data of each sample to support data records and indicate each samples provenance and experimental manipulations performed as well as the resulting data outputs and which archived records they form.

## Technical Validation

The 3D imaging techniques (OPT and LSFM), image processing pipelines and experimental procedures used to generate the presented data have been rigorously tested and have been evaluated in peer reviewed journals (see e.g., refs. ^2,6,14,16–18,20–24^). For OPT imaging we implemented a custom build setup^16^ to obtain a higher signal-to-noise ratio with the use of near-infrared filters. To achieve optimal sample alignment a custom written code to align samples pre-scan was implemented^17^. Validation of OPT scans on islets of Langerhans distribution in the entire mouse pancreas was performed in previous studies by comparing 3D scanned optical tomographic sections with confocal or 2D histological sections^6,25^. High resolution LSFM scanned regions of interest, obtained post-OPT scanning, were validated by cross referencing spatial coordinates of islet of Langerhans between OPT and LSFM scans of the same specimen. Phenotypic validation of STZ-induced diabetes was performed by blood glucose analysis and blood sampling. To ensure reliable and unbiased data production all samples were blinded and randomised at organ harvest and throughout tissue processing, tissue clearing, 3D imaging, image processing, Imaris volume quantification and statistical analysis.

## Usage Notes

Raw image projections (*.tif) and tomographic sections (*.bmp or *.ome.tif) can be converted and imported into most 3D visualisation/quantification software such as ImageJ (NIH, USA), Arivis (Munich, Germany), Imaris (Bitplane, UK) or 3D slicer (https://www.slicer.org/). When importing multiple channels generated by OPT or LSFM the numerical value of the starting image of the projection views (OPT) or z-stacks (LSFM) should always be the same for the anatomy, insulin or GLUT2 channels.

The Imaris files (*.ims) contain all iso-surfaces (anatomy, insulin or GLUT2) used for quantification and numerical data mining and can be visualised for free using the Imaris viewer (https://imaris.oxinst.com/imaris-viewer).

## Supplementary information

Supplementary Figures

Table S3

Supplementary Table S1

Supplementary Table S2

Table S4

## Data Availability

Custom generated scripts used for processing OPT data including COM-AR^17^ (alignment of axis of rotation during OPT scan setup), DFTA^17^ (alignment of axis of rotation post-OPT scanning) and CLAHE^2^ (improving islet segmentation) is compiled as a software package (together with video instructions on their implementation) at GitHub, Link “https://github.com/ARDISDataset/DSPOPT”.
