# Supplementary material for: Quantitative 3D OPT and LSFM datasets of pancreata from mice with streptozotocin-induced diabetes
Source: Sci Data. 2022 Sep 10;9:558. doi: 10.1038/s41597-022-01546-5 (PMC9464185; doi:10.1038/s41597-022-01546-5)
Supplement: Supplementary file 1 — Supplementary Figures [file 41597_2022_1546_MOESM1_ESM.pdf]

Supplementray Figures

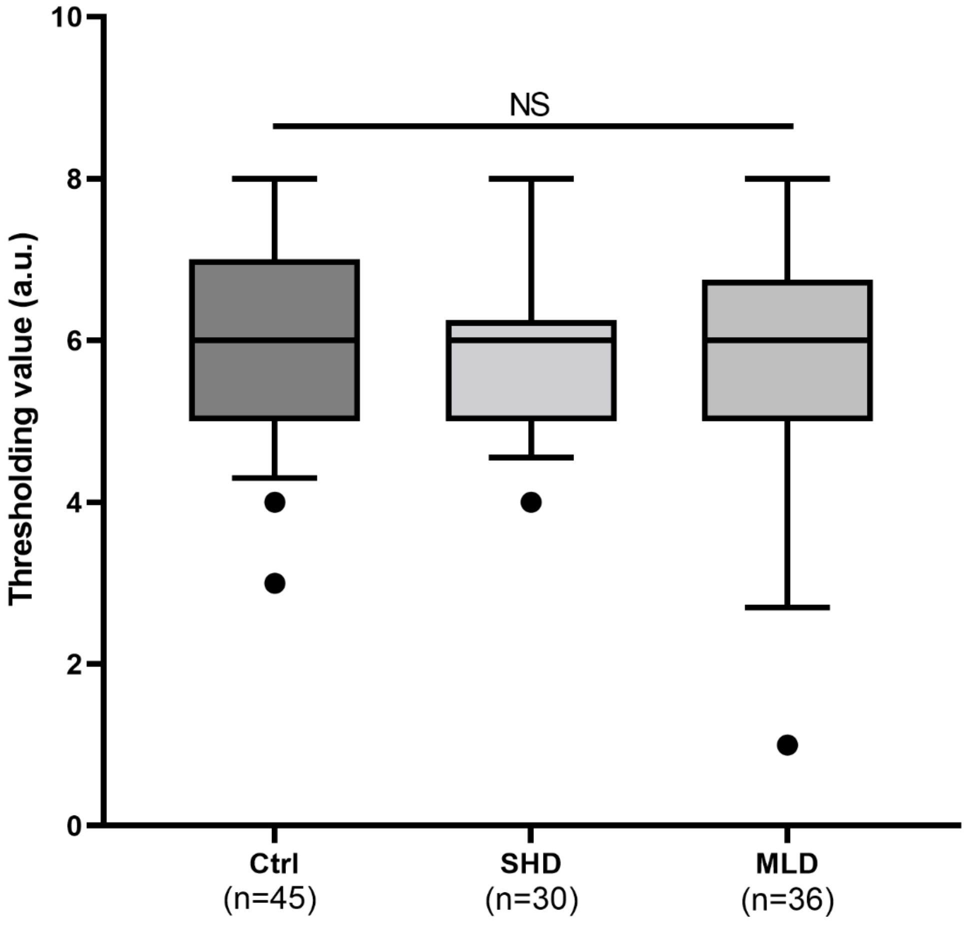

Supplementary Figure 1: Box and whiskers graph depicting thresholding values based on islet volume segmentation for quantification in Imaris. Ctrl, SHD and MLD is not significantly different (p = 0.2524 using a non-parametric one-way ANOVA (Kruskal-Wallis) test). Individual values can be found in Supplementary table 1 and 2.
